# Bilateral Upper Extremity Tremors in West Nile Encephalitis

**DOI:** 10.7759/cureus.37168

**Published:** 2023-04-05

**Authors:** Kaitlin J Crum, Rainier Batiste

**Affiliations:** 1 Internal Medicine, Edward Via College of Osteopathic Medicine, Hammond, USA; 2 Internal Medicine, North Oaks Medical Center, Hammond, USA

**Keywords:** west nile virus infection, acute encephalitis, viral infection, neuroinvasive west nile virus, movement disorders and tremors, west nile encephalitis

## Abstract

West Nile encephalitis is a rare complication of infection from the West Nile virus (WNv). Viral encephalitis can mimic manifestations of other neurologic diseases. The purpose of this article is to report a case of a 60-year-old female who developed bilateral upper extremity tremors with West Nile encephalitis. She presented to a hospital in Southern Louisiana with persistent high fevers and new onset confusion. She soon developed tremors which persisted throughout her hospitalization. Computerized tomography (CT) of the head revealed no abnormalities. Cerebral spinal fluid (CSF) was remarkable for WNv IgM, and supportive care was pursued. After nearly three weeks, she was transferred to a skilled nursing facility for further care.

The presentation of movement disorder with confusion usually raises concern for injury to the brain or spinal cord or other neurologic illnesses. Despite the presentation of movement disorders or other neurologic manifestations, viral etiologies should remain high on the differential when the patient has additional symptoms, such as fever and elevated white blood cell (WBC) count, to limit inappropriate diagnostic testing and treatment.

## Introduction

West Nile encephalitis is caused by a virus transmitted to humans by* Culex *spp. mosquitoes [1]. A mosquito becomes infected by biting an infected bird and can then spread the virus by biting other animals or humans [2]. Unlike other viruses, West Nile virus (WNv) is not transmitted through respiratory droplets; however, it can be spread through blood products and breast milk [2]. Endemic to Africa and the Middle East, WNv was first reported in North America in 1999 [3]. While most people with the virus are asymptomatic, about 20% present with a fever and flu-like symptoms of headache, body aches, vomiting, and fatigue that subside within a week [4]. Only about 0.67% will develop a severe disease such as encephalitis or meningitis, and age is the most important risk factor for this development [1]. Of adults over age 60 infected with the virus, about 2% develop neurologic diseases, and 10% of people of any age with severe illness affecting the central nervous system die [4]. Diagnosis is made by the detection of IgM in blood or cerebral spinal fluid (CSF). IgM antibodies may persist for 30-90 days [5]. WNv has no specific treatment or vaccine, and hospitalization is often not required but may be necessary for symptomatic relief of severe pain and dehydration [6].

The case reported here involves a 60-year-old female who presented to the hospital with persistent fever and acute onset confusion. Substantial tremors of the upper extremities developed, which raised concern for neurologic disease. Workup was significant for West Nile IgM in the CSF sample. The patient’s fever and confusion subsided, but tremors remained throughout the hospitalization.

## Case presentation

A 60-year-old Caucasian female, with a chronic smoking history and past medical history of chronic obstructive pulmonary disease, presented to the emergency department with acute confusion, slurred speech, nausea, and a temperature of 103.3 F. The patient’s symptoms started a few days prior with a low-grade fever, and an at-home COVID-19 test was negative. Other vital signs were significant for oxygen saturation of 91% on room air and tachypnea at 22 breaths per minute. The patient was confused and had difficulty answering questions. There was no known history of recent insect bites or toxin exposure. Physical exam was negative for rashes, neck pain, or suprapubic tenderness, and cranial nerves were intact.

No abnormalities were noted on non-contrast computerized tomography (CT) of the head or chest radiograph. Initial laboratory studies were significant for leukocytosis of 13.6 x 10^3^/μL with an elevated absolute neutrophil count of 12.0 x 10^3^/μL and microcytic anemia. Urinalysis indicated trace ketones and moderate blood in the urine. The urine drug screen was negative. The patient denied a history of chronic Foley catheter use, dysuria, urinary tract infections, or pyelonephritis. A viral panel and blood cultures were obtained, and empiric antibiotics of cefepime and vancomycin were started. She was given a bolus of normal saline and ondansetron for relief of nausea. As her fever was controlled with antipyretics, her mental status began returning to baseline. She was admitted to the medicine unit.

On day two of hospitalization, the patient continued to have high fevers and slurred speech. Significant tremors in the upper extremities were noted on a physical exam that prompted a neurology consult. CT of the head was repeated with no abnormalities noted. Although no seizure activity was noted on the electroencephalogram (EEG), levetiracetam was added to the patient’s treatment regimen. Infectious disease was consulted. Intravenous acyclovir was added to the regimen due to a history of herpes simplex virus (HSV). Lumbar puncture showed elevated white blood cells (WBCs), protein, and decreased glucose in CSF. CSF was screened for antibodies of various infectious agents including WNv, herpesviruses 1 and 2, varicella-zoster virus, and *Treponema pallidum*.

On day three, the patient continued experiencing severe tremors in her upper extremities bilaterally. Strength was diminished, but sensation remained intact. A repeat EEG was positive for seizure activity, and the patient remained on levetiracetam. The fever spiked overnight to 102.4 F, and the patient’s confusion persisted. The next day, her fever subsided. WBC counts normalized, as shown in *Table 1*, and remained normal for seven days.

**Table 1:** Patient’s WBC Trend Reference range and units: 4.4-11.2 x 10^3^/μL WBC, white blood cell

Day #	1	2	3	4	11	12	13	14	15	16	17
WBC	13.6	16.9	14.1	9.9	10.3	11.3	13.1	16.2	14.8	11.7	11.0

Over the next week of her hospitalization, her body temperature remained normal, and her mental status significantly improved. She was able to follow commands and communicate with her family. CSF analysis indicated West Nile IgM, and no other causative agents were detected. Due to the patient’s confusion and seizure activity, the diagnosis was amended to West Nile encephalitis. Antibiotics and antivirals were discontinued, and conservative treatment was pursued.

On day 11, WBC counts began to rise reaching a peak of 16.2 x 10^3^/μL on day 14 before normalizing again (Table 1). Occupational and physical therapists recommended placement in a skilled nursing facility due to her persistent tremors and weakness. After two weeks of in-patient care, she began experiencing hallucinations and delusions. Psychiatry was consulted for evaluation and treatment. Psychosis resolved on day 17, and the decision was made to reduce the patient’s levetiracetam dosage due to its potential side effect of hallucinations. After 18 days of hospitalization, the patient was discharged to a skilled nursing facility for further supportive therapy.

## Discussion

This patient presented with bilateral tremors in the upper extremities due to West Nile encephalitis. Encephalitis was consistent with persistent high fever, seizures, tremors, and altered mentation. CSF revealed pleocytosis and West Nile IgM. The majority of people infected with the virus are asymptomatic, and less than 1% of people develop neurologic infections such as encephalitis [1]. In a study of 16 patients with viral encephalitis, 15 of them presented with a tremor like the one presented in this case. Other movement disorders, such as myoclonus and parkinsonism, may be present during the acute phase [7]. Rarely, WNv can cause acute flaccid paralysis which can mimic Guillain-Barre syndrome or poliomyelitis [8]. Other neurologic complications have also been reported, such as acute transverse myelitis [9]. These reports indicate that WNv can mimic other neurologic diseases, making it difficult to diagnose apart from the detection of IgM in CSF or serum. The differential diagnoses, in this case, included cerebral infarct, sepsis due to bacterial infection, and HSV viral meningitis, which led to diagnostic imaging studies and the initiation of broad-spectrum antibiotic therapy and antiviral therapy. Upon receival of lumbar puncture and CSF analysis results, treatment was de-escalated to provide supportive care only. Immunoglobulin therapy has been associated with shortening the course of the illness when given early in the disease progression but was not pursued in this case due to the patient's late presentation [10]. The CDC recommends treatment for WNv to include rest, fluids, and pain medication for symptomatic relief [4]. Although there are human WNv vaccine candidates in ongoing clinical trials, currently none have been approved [11]. Additionally, patients presenting with movement disorders are likely to benefit from occupational and physical therapy. Likewise, in one observational retrospective study, researchers found that in-patient rehabilitation resulted in significant recovery of functional status in patients with the severe neuroinvasive disease [12]. After nearly three weeks of hospitalization, the patient in this case was transferred to a skilled nursing facility for in-patient rehabilitation.

## Conclusions

In conclusion, this case underlines a rare symptom of tremors in West Nile neuroinvasive disease. It serves to reinforce the importance of keeping viral encephalitis on the differential when a patient presents with movement disorders accompanying high fever and leukocytosis. Recognition that such presentations may be of viral origin may help avoid inappropriate treatment and diagnostic testing.
